# Endometrial Tumor Microenvironment Alters Human NK Cell Recruitment, and Resident NK Cell Phenotype and Function

**DOI:** 10.3389/fimmu.2019.00877

**Published:** 2019-04-26

**Authors:** Clara Degos, Mellie Heinemann, Julien Barrou, Nicolas Boucherit, Eric Lambaudie, Ariel Savina, Laurent Gorvel, Daniel Olive

**Affiliations:** ^1^Tumor Immunology Team, IBISA Immunomonitoring Platform, Cancer Research Center of Marseillle, INSERM U1068, CNRS U7258, Institut Paoli-Calmettes, Aix-Marseille University, Marseille, France; ^2^Department of Surgical Oncology 2, CNRS, INSERM, Institut Paoli-Calmettes, CRCM, Aix-Marseille University, Marseille, France; ^3^Institut Roche, Boulogne Billancourt, France

**Keywords:** endometrial cancer, NK cells, Tigit, Tim-3, resident cells, immune checkpoint

## Abstract

Endometrial Cancer is the most common cancer in the female genital tract in developed countries, and with its increasing incidence due to risk factors such as aging and obesity tends to become a public health issue. However, its immune environment has been less characterized than in other tumors such as breast cancers. NK cells are cytotoxic innate lymphoid cells that are considered as a major anti-tumoral effector cell type which function is drastically altered in tumors which participates to tumor progression. Here we characterize tumor NK cells both phenotypically and functionally in the tumor microenvironment of endometrial cancer. For that, we gathered endometrial tumors, tumor adjacent healthy tissue, blood from matching patients and healthy donor blood to perform comparative analysis of NK cells. First we found that NK cells were impoverished in the tumor infiltrate. We then compared the phenotype of NK cells in the tumor and found that tumor resident CD103^+^ NK cells exhibited more co-inhibitory molecules such as Tigit, and TIM-3 compared to recruited CD103^−^ NK cells and that the expression of these molecules increased with the severity of the disease. We showed that both chemokines (CXCL12, IP-10, and CCL27) and cytokines profiles (IL-1β and IL-6) were altered in the tumor microenvironment and might reduce NK cell function and recruitment to the tumor site. This led to hypothesize that the tumor microenvironment reduces resident NK cells cytotoxicity which we confirmed by measuring cytotoxic effector production and degranulation. Taken together, our results show that the tumor microenvironment reshapes NK cell phenotype and function to promote tumor progression.

## Introduction

Endometrial cancer is the most common cancers of the female genital tract in developed countries. Despite a good survival overall (70% of 5-year survival), prognosis associated with a decreased survival rates (<20% of 5-year survival) are observed in patients with late stages of endometrial cancer. Furthermore, endometrial cancer incidence tends toward increasing as risk factors risk factor such as obesity and aging are involved. These factors, associated with exogenous estrogen exposure contribute to the spreading of the disease in western countries and might lead to a major public health issue (1, 2).

Histological studies showed that endometrial cancer could be separated in two groups based on histological characteristics. The first group is composed of endometrioid adenocarcinoma, while the second group regroups all the others histology which include carcinosarcoma, clear cell carcinoma, serous adenocarcinoma. Type I endometrial cancers are subdivided following 3 grades, which are associated with the differentiation status of cells ranging from the most differentiated to the less differentiated, which are associated, respectively with a good and a bad prognosis. Type II endometrial cancers are usually associated with a poorer survival than Type I (3, 4).

Endometrial cancers are also separated and classified according to the tumor localization. This anatomical classification designed by the FIGO status is used, along with the histological analysis and the TNM system from the American Joint Committee on Cancer [the extent of the tumor (T), the invasion of lymph nodes (N), the spreading of distant sites (M)] to define the treatment (5). In addition to that, endometrial cancer has been less studied than other tumors such as breast, lung or colorectal cancer, and therefore some aspects of this disease remain poorly understood. This is the case of the Immunology of the endometrial tumor environment. Only few reports show that the immune system is involved in the regulation of endometrial tumor progression. Kondratiev et al. showed that the presence of CD8^+^ T cells inside the endometrial tumor represents a marker of patient survival (6). Similarly, in another study it was demonstrated that intraepithelial CD103^+^ CD8^+^ tumor infiltrating T cells expressed PD1^+^, were antigen-activated and were associated with improved survival (7).

However, The Cancer Genome Atlas (TCGA) recently sequenced and identified several genomic endometrial cancer patient profiles (8). Indeed, 4 genetic profiles were found and used to stratify the patients with different survival, interestingly the POLE (polymerase ε) mutated profile correlated with the best survival. Some studies showed that this POLE mutated profile is associated with a high CD8^+^ T cells infiltration, PD-1 expression on TILS, and also CD4^+^ T cell responses (8–10). This highlights the importance of tumor infiltrating leukocytes in the pathophysiology of endometrial cancer.

Natural Killer cells (NK cells) are cytotoxic innate lymphoid cells that are a major anti-tumoral effector cell type along with CD8^+^ T cells and γδT cells.

NK cell activation and cytotoxicity relies on a balance between inhibitory and activating signaling. For instance, class I Human Leukocyte Antigens, Ig like transcripts, Tigit (T cell Immunoreceptor with Ig and ITIM domain), inhibitory cytokines (TGF-β, IL-6, IL-32a) provide inhibitory signals for NK cell functions and activation. On the contrary, various activating receptor engagement, such as DNAM1, KIRs, NKp80, NKG2D, and NCRs, along with activating cytokines (IL-15, IL-18, IL-12) lead to an activation of NK cell (11–18). Indeed, activating receptors are triggered by stress-induced, self-molecules, and viral components. In cancers, NK cells are efficient during the elimination phase as they control tumor growth. However, NK cell function is often altered during tumor immune evasion which allows tumor growth and tissue invasion.

Indeed, in colorectal cancer, NK cells infiltration is correlated with a better prognosis and outcome (19–21), in lung cancer NK cells displayed an inhibitory phenotype as there is a downregulation of the expression of the NKp30 and NKG2D, their cytolytic function is also impaired by a reduced production of Granzyme-B (GrzB) (22, 23). Finally, in breast tumors, reports showed that breast cancer cells increase self-tolerance by modifying NK cell phenotype and were unable to repress tumor growth (24).

Therefore, we investigated NK cell profiles in endometrial tumors. For that, we gathered endometrial tumors, tumor adjacent healthy tissue, blood from matching patients, and healthy donor blood to perform comparative analysis of NK cells. First, we found that there were very few NK cells in the tumor infiltrate and that the amount of CD56^bright^ NK cells was increased in the tumor. We then compared the phenotype of NK cells in the tumor and found that tumor resident NK cells (expressing CD103) exhibited more co-inhibitory molecules such as T cell Immunoreceptor with Ig and ITIM domain (Tigit), and T cell immunoglobulin and mucin domain containing 3 (Tim-3) compared to non-resident NK cells. Furthermore, IL-15, a pro-cytotoxic cytokine, was reduced, whereas IL-6, an inhibitor of the STAT-5 pathway and of the NK cell function (25), was increased in the tumor. This led to hypothesize that tumor resident NK cells lost their cytotoxic function. Thus, we tested cytolytic effector production by NK cells and we observed that they were reduced in the tumor, which was confirmed by functional assays. Here we could demonstrate that the endometrial cancer tumor microenvironment is of great influence on resident NK cells as it could reduce their cytotoxic capacity and therefore promotes tumor progression.

## Materials and Methods

### Patients and Ethics

Patients were included in the Paoli Calmettes Institute “GC Bio” clinical trial (NCT01977274) which aims at characterizing gynecological cancers. The GC-Bio protocol inclusion process will last 5 years and the patient's follow up will be done over 10 years. This study has been accepted by the national ethics committee (ANSM, Agence Nationale de Sécurité du Médicament, n°130995B-12 and CPP, Comité de Protection des Personnes, n° CPP 13 62). The registration number of the study is ID-RCB: 2013-A00992-43.

Our cohort includes patients with endometrial cancer prior to any treatment. The lymph nodes invasion assessment was performed using imaging (MRI, CT scan or PET scan) and, when required, pelvic and/or para-aortic lymphadenectomy. Patient blood samples were collected before the initial surgery. A tumoral sample, along with an adjacent non-invaded endometrial tissue sample (assessed by the pathologist macroscopically), were resected during the initial surgery and before any other treatment. The healthy blood was obtained from French Blood Bank (Etablissement Français du Sang-EFS).

### Cell Isolation

The tissue samples were weighted, and dissociated manually using scalpels in RPMI 1640 (Gibco). The cells were isolated after filtration on 70 μm then 30 μm filters and centrifuged (300 × *g* for 5 min at 4°C). Tissue supernatants were kept to quantify the cytokines released in the tissue microenvironment. PBMCs were isolated using a Ficoll gradient (Eurobio). Briefly, whole blood was diluted by adding an equal volume of PBS, deposited slowly onto Ficoll media and centrifuged at 800 × *g* for 30 min at room temperature with no break or acceleration. Cells were recovered from the interface with the plasma, washed twice in PBS, then counted and prepared for the experiments. Serum and plasma were also collected and frozen at −80°C before use to allow the quantification of circulating cytokines and chemokines.

### Flow Cytometry

Isolated cells were centrifuged, and then stained for 20 min at 4°C in the dark with various mixes of antibodies (listed in Supplementary Table 1) in brilliant stain buffer (BD Biosciences), after a wash in PBS, we stained the cells with a viability marker [LIVE/DEAD Aqua (Life Technologies)] for 20 min at 4°C in the dark. For intracellular staining, we used BD Biosciences Cytofix/Cytoperm kit, according to manufacturer's instructions. Briefly, after the extracellular staining, cells were permeabilized in Fixation/Permeabilization solution for 20 min at 4°C, cells were then washed twice in Permwash buffer before intracellular staining during 20 min at 4°C. Appropriate isotype antibodies were used as controls. The entire tube of cells was then acquired on a FACS LSR2 (Becton Dickinson). To assess the absolute cell number we used True-count beads (BD Bioscience). Application settings and sphero rainbow beads (BD Biosciences) were used to ensure reproducible and comparable results between patients and over time. BD DIVA software was used for data acquisition and FlowJo (Treestar) software was used for the analysis.

### Functional Assays

Tissue cells (from tumor and non-invaded endometria) were plated in 96 well-plates in RPMI 1640, 10% FCS, 1% of Penicillin/Streptomycin (Gibco), 200 UI/ml of IL-2 (Proleukine) at 37°C with 5% CO_2_. After 16 h, we added PMA (Sigma Aldrich, 25 ng/ml), Ionomycin (Sigma Aldrich, 1 μg/ml), GolgiStop (BD Biosciences, 0.4 μl/200 μl), anti-CD107a and anti-CD107b FITC antibodies (BD Biosciences) in the wells and cells were incubated for 6 h at 37°C and 5% of CO_2_. Cells were then harvested and stained for both extracellular markers and intracellular cytokines, as described above.

### Cytokine and Chemokine Quantification

Human ProcartaPlex Mix & Match assays (eBiosciences/Life Technologies) and the 40 plex Bio-Plex Pro™ Human Chemokine Panel mix were used to test the presence of chemokines and cytokines of interest. The assay was performed according to manufacturer's instructions. Plates were read on the Bio-Plex analyzer (Biorad). We used Bio-Plex Manager (Biorad) to generate standard curve and results, according to manufacturer's instructions. Results were normalized against tissue weight.

### Softwares and Statistical Analysis

GraphPad Prism was used to generate graphical results and statistical analyses. Mann-Whitney test (non-parametric *t*-test), or Wilcoxon (paired *t*-test, non-parametric) were used when paired series of tissues were used in the experiment (e.g., same patient adjacent tissue + tumor or tumor from same patient + Ec PBMC). Differences were considered significant with *p* ≤ 0.05.

## Results

### NK Cell Infiltrate Is Altered in the Tumor Microenvironment

The immune infiltrate of tumor and adjacent tissue samples as well as PBMC cell composition was assessed by flow cytometry. After gating alive and singlets cells on the size (FSC) and granularity (SSC), we could identify NK cell subtypes, CD3^−^CD56^dim^ cells and CD3^−^CD56^bright^ cells (Supplementary Figure 1) among the immune cells (CD45^+^).

**Figure 1:**
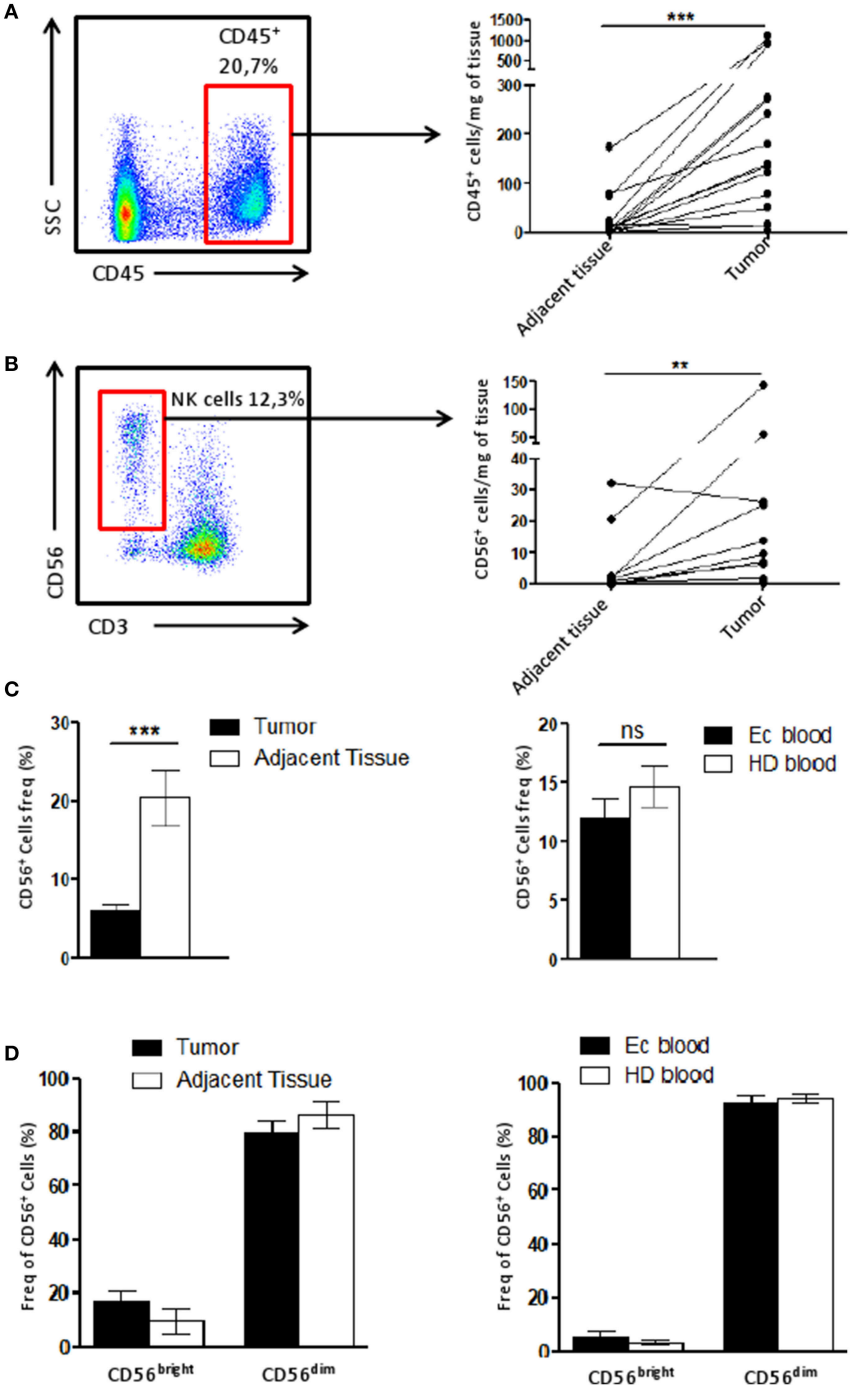
NK cell infiltrate is altered in the tumor microenvironment. **(A)** The number of CD45^+^ cells recovered per mg of either the tumor or the adjacent tissue (*n* = 15) was plotted here. Briefly, cells were counted by flow cytometry using beads. Each dot-line represents one patient. **(B)** The number of CD56^+^ CD3^−^ cells (among CD45^+^ cells), corresponding to NK cells, recovered per mg of either the tumor or the adjacent tissue (*n* = 11) was plotted here. Each dot-line represents one patient. **(C)** On the left panel, the frequency of NK cells (CD56^+^ cells) among CD45^+^ cells in the tumor (*n* = 29), in black, was compared to their frequency in the adjacent tissue (*n* = 12), in white. On the right panel, the proportion of NK cells among PBMCs from either healthy donors patients [HD blood (*n* = 13), in white bar] or endometrial cancer patients [EC blood (*n* = 18), in black bar]. **(D)** CD56^bright^ and CD56^dim^ cells were identified by flow cytometry among the NK cell population in tissues on the left panel (tumor, *n* = 13, in black and adjacent tissue, *n* = 6, in white), and for HD (white bar, *n* = 7) and EC blood (black bar, *n* = 4) on the right panel. Mean ± SEM of different patients' samples, **p < 0.01, ***p < 0.001, ns: not significant (*p* > 0.05).

To identify the immune infiltrate in the tissue, the number CD45^+^ cells found per mg of tissue was calculated and we showed that the immune infiltrate in the tumor was higher compared to that of the adjacent tissue (Figure 1A). Using the same quantification method, we demonstrated the NK cell infiltration increased along with the CD45^+^ in the tumor compared to the matching adjacent healthy tissue (Figure 1B).

We then described the proportion of NK cells among the CD45^+^ population. Tumor NK cells represented 6.1 ± 1% of CD45^+^ cells while in the adjacent tissue they represented 20.4 ± 4% of immune cells (Figure 1C). This suggests that the immune infiltrate in the tumor is altered compared to adjacent tissue. Interestingly, blood samples from either patients (EC blood), or healthy donors (HD blood) harbored a similar quantity of NK cells among CD45^+^ cells (Figure 1C). Note worthily, no differences were observed for the profile of NK CD56^bright^ or CD56^dim^ cells between tumor and adjacent tissue, or between blood from patients or healthy donors (Figure 1D).

### Resident NK Cells Exhibit an Immunoregulatory Phenotype

**Figure 2:**
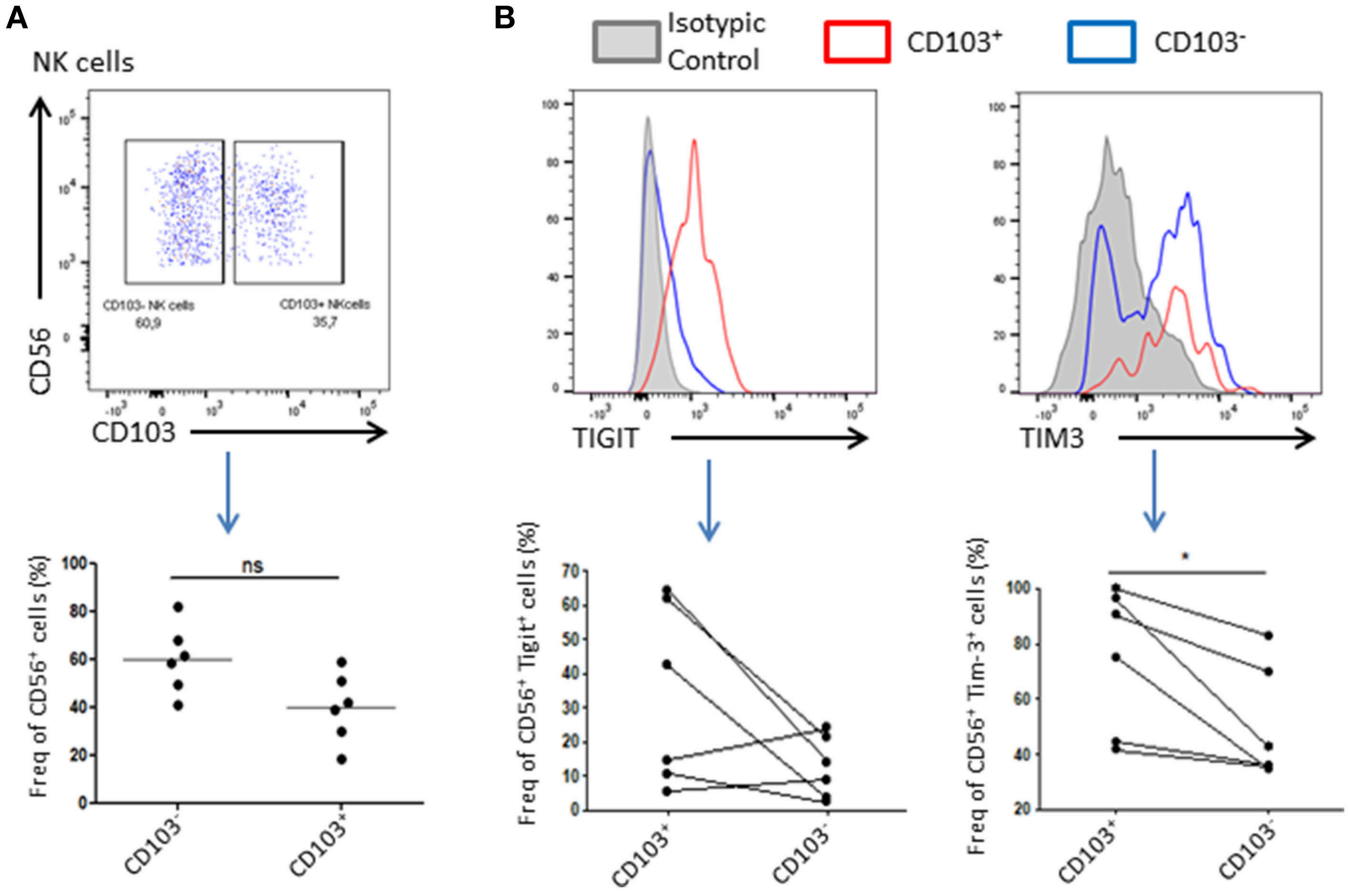
Resident NK cells displayed an inhibitory profile in the tumor. **(A)** Resident NK cells (CD45^+^CD3^−^CD56^+^) were identified based on CD103 expression. A representative dot plot of CD103^+^ and CD103^−^ NK cells gating is shown. Below, a plot chart of CD103^+^ and CD103^−^ NK cells frequency among all CD56^+^ cells is shown, where each dot represents one patient (*n* = 6). **(B)** Upper panel: Representative graphs of the expression level of Tigit (left panel) or Tim-3 (right panel) in CD103^+^ NK cells (in red) or CD103^−^ NK cells (in blue). The isotype control was represented in gray. The positive gate was made using the isotype control (>1% of isotype control staining was considered as positive). Lower panel: The expression of Tigit (left panel) and Tim-3 (right panel) among NK cells CD103^+^ or CD103^−^ was assessed and plotted here. Each dot-line represents one patient *n* = 6. *p < 0.05, ns: not significant (*p* > 0.05).

As NK cells abundance is reduced in tumor compared to healthy adjacent tissue, we wondered if their phenotype might be affected by the tumor microenvironment. For this purpose, we added a CD103 staining to our panel that allowed us to discriminate resident cells and recruited cells. Indeed, CD56^+^CD103^+^ cells represent a population of resident NK cells whereas CD56^+^CD103^−^ represent their recruited counterpart (Supplementary Figure 1). We found that 40 ± 19% of total NK cells expressed CD103 and were likely to be tumor resident NK cells. Tigit and Tim-3 are usually associated, in cancer, with an exhausted phenotype of immune cells, and weaker immune response as well as anti-inflammatory and anti-cytolytic responses. Indeed, we could observe a trend in Tigit expression, which seemed to increase in CD103^+^ NK cells, and a significant increase in the expression of Tim-3 in these same cells compared to CD103^−^ NK cells, suggesting an inhibitory environment prone to abolish the cytotoxic effect of NK cells (Figure 2B).

### NK Cells Co-inhibitory Molecule Expression Is Higher in Patients With Lymph Node Invasion

**Figure 3:**
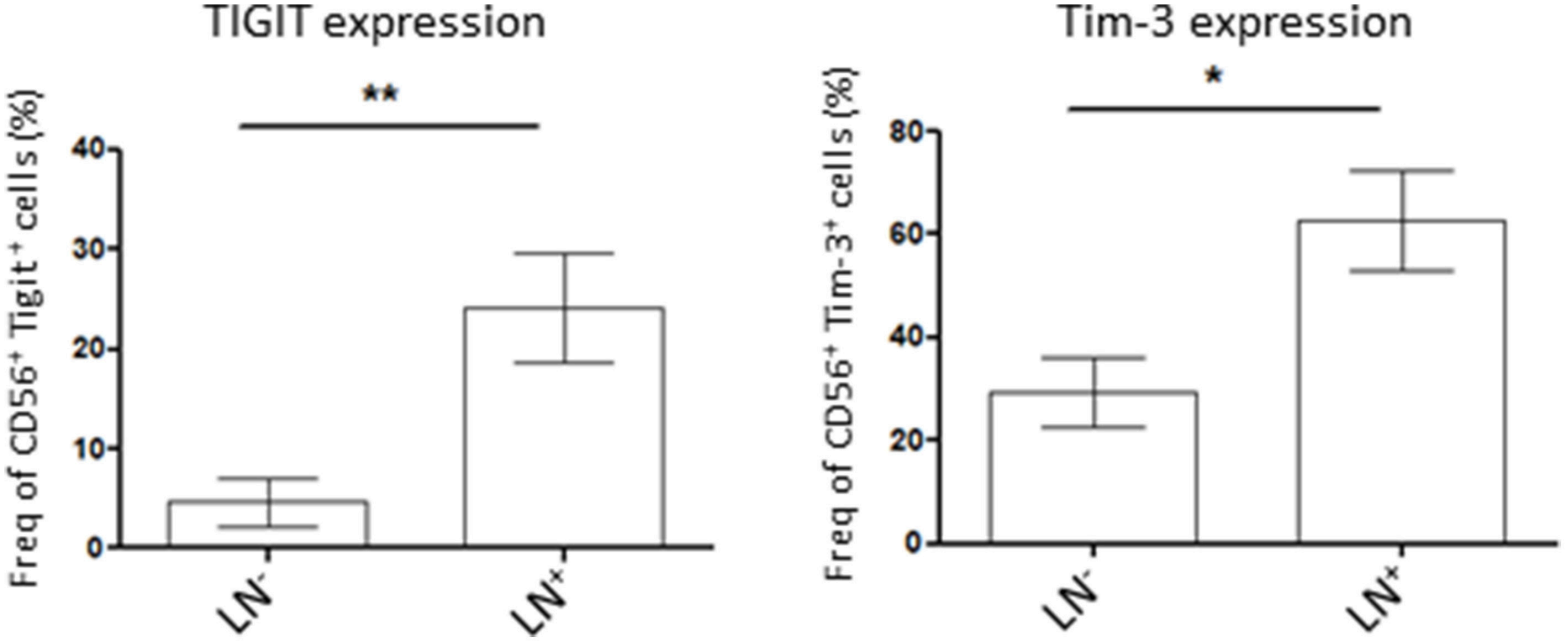
Patient lymph nodes (LN) invasion is associated with higher expression of co-inhibitory molecules in tumor NK cells. Isolated tumor cells were stained for Tigit (left, *n* = 7 for LN^−^ patients and *n* = 5 for LN^+^ patients) or Tim-3 (right, *n* = 13 for LN^−^ patients and 5 for LN^+^ patients) by flow cytometry and their expression was assessed in NK cells (CD56^+^ cells). Their expression was compared among patient with lymph nodes invasion (LN^+^) or not (LN). Mean ± SEM of different patients' samples, *p < 0.05, **p < 0.01.

Lymph node (LN) invasion in endometrial cancer correlates with advanced tumors stages and therefore, poorer outcome for the patient. As we demonstrated that tumor NK cells showed variability in the expression of the co-inhibitory molecules Tigit and Tim-3 we wondered if their expression was correlated to the severity of the disease. For that, we compared Tigit and Tim-3 expression on tumor NK cells from LN-invaded or uninvaded patients. We showed that NK cells in patient harboring LN invasion had a significant increase in Tigit or Tim-3-expressing NK cells compared to patient with no LN invasion (24.2 ± 6% in LN^+^ vs. 4.6 ± 2% in LN^−^ and 62.8 ± 12% in LN^+^ vs. 29.5 ± 5% in LN^−^, respectively) (Figure 3). Taken together, these results demonstrate that NK cells immunosuppressive phenotype increases with lymph node invasion and therefore, the severity of the disease.

### NK Cells Are Shaped by the Chemokines and Cytokines Environment

**Figure 4:**
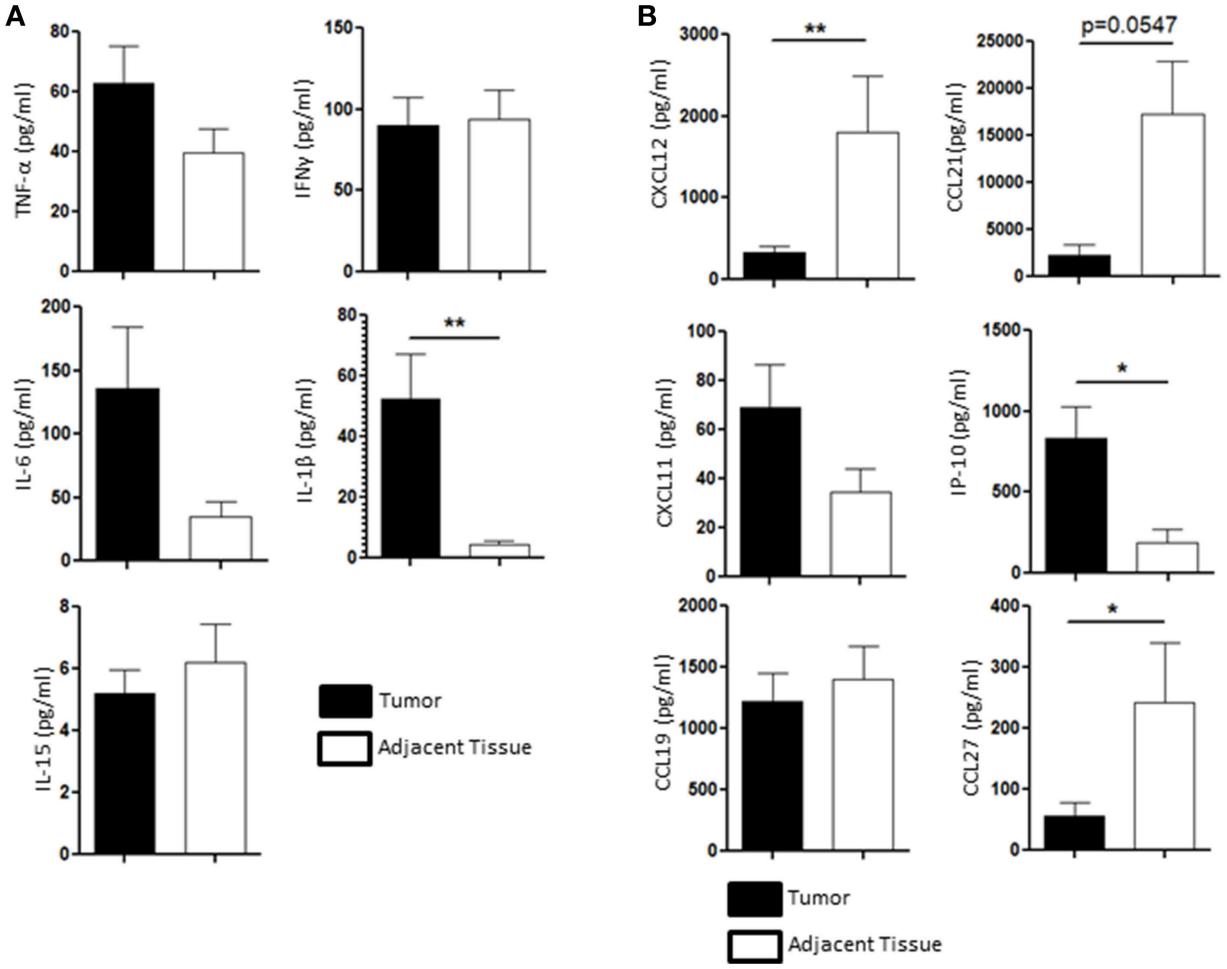
Cytokines and chemokines profile is different between the tumor and the healthy adjacent tissue, regulating NK cells differently. **(A)** The presence of TNF-α, IFN-γ, IL-6, IL-1β, and IL-15 in the supernatant of the tissue dissociation was assessed by Luminex and compared between the tumor (black bars, *n* = 11) and the healthy adjacent tissue (white bars). **(B)** The presence of the following chemokines in the tumor microenvironment (black bars) was compared to the ones found in the healthy adjacent tissue (white bars, *n* = 11): CXCL12, CCL21, CXCL11, IP-10, CCL19, and CCL27. Mean ± SEM of different patients' samples, *p < 0.05, **p < 0.01.

We then wondered whether the chemical tumoral microenvironment, and more specifically the cytokines and chemokines present in the tissue, could play a role in the NK cell homeostasis. We performed multiplex immuno-assay (Luminex) to assess the presence of inflammatory cytokines involved in the regulation NK cells function, such as TNF-α, IFN-γ, IL-6, IL-15, and IL-1β, in the tumor microenvironment (black bars, Figure 4A) and in the healthy adjacent tissue microenvironment (white bars, Figure 4A). IL-1β, a pro inflammatory cytokine that participates to both cytotoxic response and tumoral angiogenesis, was the only cytokine that was significantly secreted in higher amount in the tumor (52.3 ± 14.8 pg/ml) compared to the adjacent healthy tissue (4.65 ± 2 pg/ml) (Figure 4A). Indeed, nor IL-15 (5.2 ± 1 pg/ml in the tumor vs. 6.2 ± 1.2 pg/ml in the adjacent tissue), TNF-α (62.7 ± 11 pg/ml in the tumor vs. 39.7 ± 9 pg/ml in the adjacent tissue) or IFN-γ (62.7 ± 18 pg/ml in the tumor vs. 93.6 ± 20 pg/ml in the adjacent tissue) were found to be significantly modulated in the tumor compared to the adjacent healthy tissue (Figure 4A). The secretion of IL-6, a negative regulator of NK function (25, 26), tended to increase in the tumor rather than in the adjacent tissue as its concentration reached 135.7 ± 50 pg/ml in the tumor compared to 34.9 ± 11 pg/ml in the adjacent healthy tissue.

We also investigated the presence of CXCL12, CCL21, CXCL11, IP-10 (or CXCL10), CCL19, and CCL27, which participate to the recruitment of specific NK cell populations to the tumor site. CCL19 and CXCL11 were not differentially secreted in the tumor (1221.47 ± 250 pg/ml and 69.1 ± 9 pg/ml, respectively) compared to the adjacent tissue (1399.5 ± 300 pg/ml and 34.6 ± 5 pg/ml, respectively). IP-10 (also known as CXCL10) was found to be enriched in the tumor (835.9 ± 210 pg/ml) compared to the adjacent healthy tissue (188.1 ± 100 pg/ml). We also found that CXCL12, CCL27, and CCL21 were found in higher quantities in the healthy adjacent tissue (1795.6 ± 700 pg/ml, 242.9 ± 95 pg/ml, and 17285.9 ± 6000 pg/ml, respectively) than in the tumor (325.8 ± 72 pg/ml, 56.7 ± 20 pg/ml, and 2313.6 ± 1,000 pg/ml, respectively) (Figure 4B). All together, we showed that both cytokine and chemokine production in the endometrial tumors may participate to the alteration of NK cell function, as IL-6 and IL-1β are increased in the tumor, and recruitment, as chemoattractants CCL27, CXCL12, and CCL21 are significantly reduced in the tumor.

### NK Cell Function Is Altered in Endometrial Cancer

**Figure 5:**
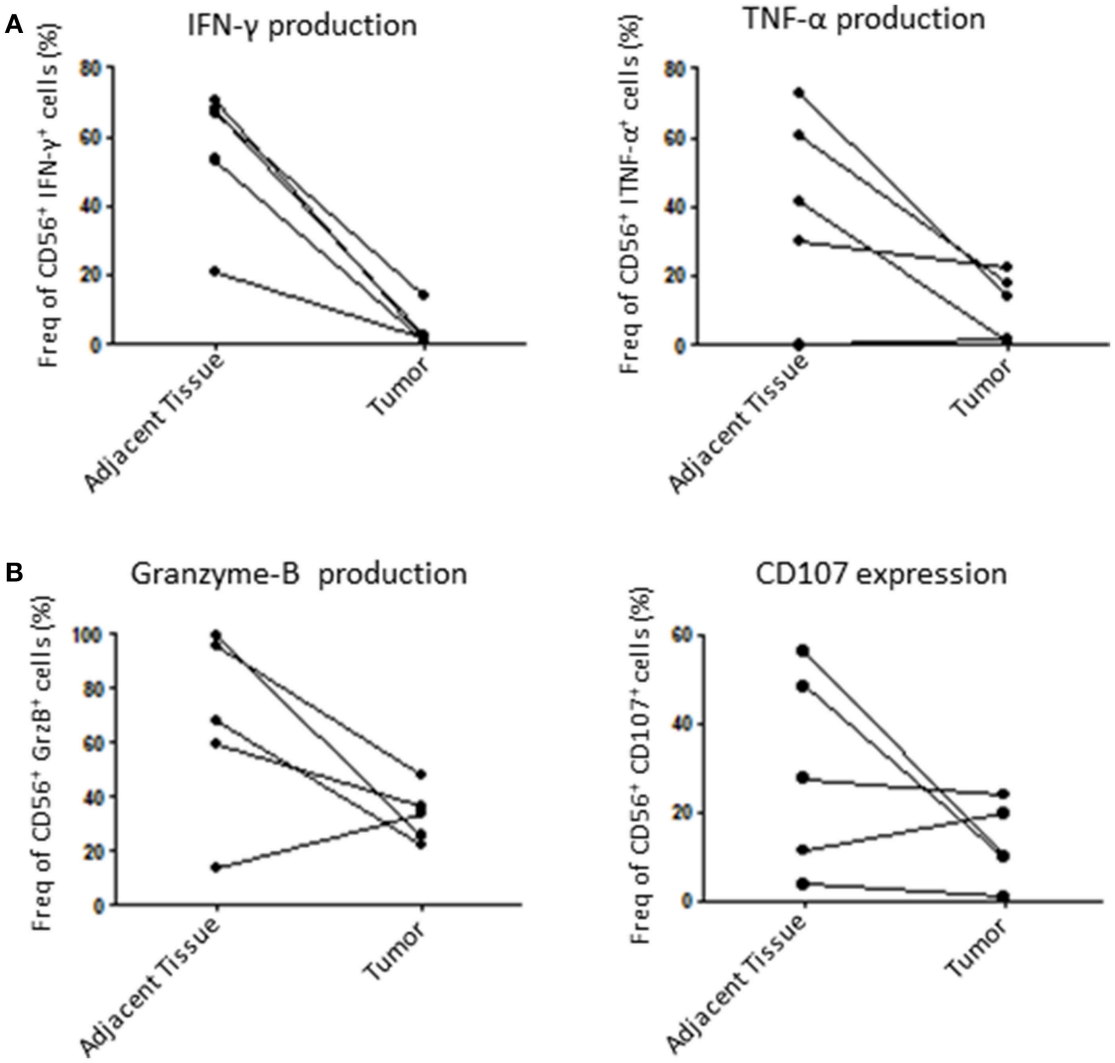
NK cells function is altered in the tumor compared to adjacent tissue. Isolated cells from either the tumor or the matching adjacent tissue were cultured overnight at 37°C. They were then stimulated with PMA/Ionomycin for 6 h and stained for intracellular cytokines. **(A)** We compared the percentage of intracellular expression of IFN-γ and TNF-α, or, **(B)**, Granzyme-B (GrzB) and CD107 in NK cells. Each dot/line represents one patient *n* = 5.

Finally, we assessed the capacity of tumoral NK cells to mount an efficient anti-tumoral response through the production of known cytolytic mediators such as GrzB (27) and IFN-γ, which can be used as cytolytic function markers (28). For that, we incubated dissociated tumors with IL-2 overnight and then stimulated them with PMA/Ionomycin to induce an immune response by measuring IFN-γ, TNF-α, GrzB production and CD107 expression (a surrogate for degranulation). Here, we could show that tumor NK cells are less responsive than healthy adjacent tissue NK cells. Indeed, tumoral NK cells produced less IFN-γ, TNF-α and GrzB and were less potent at degranulation in 4 out of 5 patients tested (Figures 5A,B). Taken together, these results strongly suggest that tumoral NK cells are less efficient than adjacent tissue NK cells to mount an anti-tumoral response in endometrial cancer.

## Discussion

Here we showed that NK cells were impoverished in the tumor immune cells population, compared to adjacent healthy tissue. Interestingly, resident NK cells also seemed to harbor inhibitory and exhaustion hallmarks such as high Tigit and Tim-3, which are associated with advanced diseases. The study of the tumoral NK cells function revealed a trend showing that NK cells seemed to be deficient at mounting a cytolytic immune response. We also showed that some of these features could depend on the surrounding microenvironment of NK cells, such as specific chemokine presence in the tumor. Indeed, CCL27 and other NK chemoattractant molecules are less produced by the tumor microenvironment than by the adjacent tissue. We showed that NK recruitment in the tumor is altered compared to the one in the adjacent tissue. Taken together these results showed that NK cells biology is deeply impaired by the tumor.

The NK cell population is one of the main anti-tumor actors of the immune system. These cells are often impaired both phenotypically and functionally in solid tumors such as breast cancer (24), melanoma (29), in colorectal cancer (30), in lung cancer (23), and various other cancers (31–33). Here, we showed that the proportion of NK cells is lower among the immune infiltrate in endometrial tumors than in the adjacent healthy tissue. The reduction of the cytotoxic immune cells infiltration, and more particularly of NK cells, is usually associated with an immunosuppressive effect of the tumor, a poorer outcome and has been widely described in solid tumors (21, 34–36).

The recruitment of immune cells, including NK cells is regulated by tissue produced chemokines, which, in our study, may explain the lack of NK cells in the tumor. Indeed, we could find that the production of various chemokines was modified in the tumor compared to the adjacent healthy tissue. IP-10, also known as CXCL10, is a chemoattractant for NK cells, and can activate NK cells leading to the lysis of tumor cells (37–39). Interestingly, we showed here that IP-10 is strongly produced in the tumor compared to the adjacent tissue, suggesting that an immune response is occurring in the tumor. Though, considering the results we observed from the functional tests, it may not be sufficient to recruit NK cells and to maintain an efficient NK cell-driven cytolytic response. CCL27, is also a NK cell chemoattractant molecule, and is, in endometrial cancer, less produced in the tumor compared to the healthy adjacent tissue. Similarly, CCL21, which has been described to enhance the recruitment of immune cells in the tumor and to participate to the establishment of a potent immune cellular response (40–42), is significantly reduced in the tumor microenvironment compared to the adjacent tissue. The lower levels of NK cell chemoattractants (CCL27, CCL21) in the tumor microenvironment may participate to the immunosuppressive mechanism of the tumor by limiting cytolytic cell recruitment.

The CXCL12-CCR4 axis has been described to promote metastasis in various cancers and should be considered as a potent target to block the disease progression (43, 44). This axis does not seem to be related to disease progression in endometrial tumors as CXCL12 is poorly secreted in the tumor microenvironment. However, CXCL12 is also required for the NK cell recruitment, and its weak production in the tumor may participate to the impoverishment of NK cells in the tumor (41). The recruitment of potent immune cells is one of the first steps of mounting a complete anti tumoral immune response. We showed here that the tumoral microenvironment could suppress the recruitment of immune effector cells by altering the production of chemokines compared to the adjacent healthy tissue.

Another important component of the tumor microenvironment is the production of cytokines. In this study we analyze the production of various pro-inflammatory cytokines such as TNF-α, IL-15, IL-6, IL-1β, and IFN-γ. While we did not see any difference of TNF-α, IL-15, and IFN-γ secretion between the tumor and the adjacent healthy tissue, we showed a trend with a higher production of IL-6 in tumor. IL-6 is known to be a pro-inflammatory cytokine that can act on several immune cells and biological features (45). Its presence in the tissue could be related to an immune response. However, several reports showed that IL-6 is also playing a pro-tumoral role (by promoting the angiogenesis, the tumor progression and metastasis) and can be linked to a bad prognosis in patients (46–48). The IL-6/JAK/STAT3 pathway is also known to negatively regulate NK cell cytolytic response, via the inhibition of the STAT5 pathway, to promote the progression of tumors by inducing a chronic inflammation (46, 49). IL-1β was also found to be highly produced in endometrial tumors. This cytokine is a hallmark of inflammation and, similarly to IL-6, plays a dual role in the tumor immunology. Indeed, it participates to both anti-tumoral, by contributing to tissue inflammation, and pro-tumoral by enhancing angiogenesis and spreading of the disease (50–53). Therefore, the elevated production of IL-6 and IL-1β in the endometrial tumors might participate to local inflammation as well as cytotoxic effector inhibition and disease progression.

Considering the anti cytolytic effect of the tumor microenvironment, we investigated tumor NK cell phenotype and function and we could find that they were deeply altered. Tim-3 and Tigit are immune inhibitor checkpoints that can suppress the cytolytic activity of NK cells. They were reported to be expressed on immune cell membranes in cancer, thus dampening the anti-tumoral response (54). In this study we showed for the first time, that tumor resident NK cells (CD103^+^) express Tigit and Tim-3, while recruited NK cells (CD103^−^) do not. This suggests that resident immune cells were more exhausted, while circulating NK cells could still harbor classical phenotype, therefore, being activated and mount an efficient immune response. Interestingly, we showed that advanced stages of endometrial cancers (which correspond to patient with LN invasion) correlates with a higher exhaustion of NK cells through the expression of Tigit and Tim-3. The increasing expression of such exhaustion markers in advanced stages of the disease makes sense as the immune response is highly inhibited, while, in early stages of the disease, immune effectors are still efficient, and thus, are able to mount an anti-tumoral response. Interestingly, it has been shown in melanoma that NK cells found in the LN metastasis showed a reduced cytotoxicity suggesting an altered function (55). Our data seem to reinforce this idea of altered phenotype or/and function of NK cells along with advance disease.

We also demonstrated that tumor NK cells had reduced production of GrzB suggesting an impaired cytolytic function compared to adjacent healthy tissue NK cells, which could correlate with their exhausted phenotype. Therefore, therapeutic strategies aiming at blocking Tigit and/or Tim-3 signals in advanced stages of cancer seem of interest, and are currently being tested in various clinical trials in association with anti-PD1 or anti-PDL1 (clinical trials: NCT03119428 and NCT03563716) and alone in phase I (clinical trial: NCT03628677). Previous reports already showed that such a strategy would help to restore a potent function of the exhausted NK cells (56, 57). Targeting these molecules in endometrial cancer could therefore, be useful to develop new therapies and restore the immune response.

Altogether we characterized the NK cells found in the tumor microenvironment of endometrial, for the first time and we showed that resident NK cells harbored an inhibited phenotype, with an impaired function. The inhibition of the NK cells response and the NK cells phenotype also seemed to be related with an aggravation of the disease. The secreted factors such as chemokines and cytokines in the tumor could participate in the establishment or not of NK cells response and we demonstrated here an alteration of their production profile in the tumor compared to the adjacent healthy tissue. The low infiltration of NK cells among the tumor immune cells should be explored and more studies on chemokines and other attracting factors could explain this. Further studies are also required to better characterize the expression of various activating and inhibiting receptors of NK cells which have been reported to be dysregulated in various solid tumors and to identify more clearly which factors are able to shape and inhibit NK cells in the tumor. Here we described and identified potential molecules and functions that were impaired in tumoral NK cells, highlighting new targets to be studied in order to restore the NK cells function in tumor.

## Ethics Statement

Patients were included in the Paoli Calmettes Institute GC Bio clinical trial (NCT01977274) which aims at characterizing gynecological cancers. The GC-Bio protocol inclusion process will last 5 years and the patient's follow up will be done over 10 years. This study has been accepted by the national ethics committee (ANSM Agence Nationale de sécurité du Médicament, n° 130995B-12 and CPP, Comité de Protection des Personnes, n°CPP 13 62). The registration number of the study is ID-RCB: 2013-A00992-43. Written informed consents were obtained from each patients that were included in this study. The healthy blood was obtained from French Blood Bank (Etablissement Français du Sang - EFS).

## Author Contributions

CD and LG conceived and planned the experiments. CD, NB, and LG carried out the experiments, CD, LG, AS, and DO contributed to the interpretation of the results. MH, JB, and EL provided patient samples. CD and LG wrote the manuscript with support from DO. CD, LG, EL, and DO conceived the original idea. DO and EL supervised the project.

### Conflict of Interest Statement

The authors declare that this study received funding from Genentech. The funder had the following involvement with the study: Genentech provided a research grant and funded CD's salary. CD was funded by INSERM transfert and Roche/Genentech. DO is a cofounder and stakeholder of Imcheck Therapeutics. DO has licensed patents to Glaxo-Smith Kline, Janssen, and Imcheck Therapeutics. DO has received research grants from Genentech, GSK, Innate Pharma and Imcheck Therapeutics. The remaining authors declare that the research was conducted in the absence of any commercial or financial relationships that could be construed as a potential conflict of interest.

## Supplementary Material

Supplemental Figure 1 NK cell gating strategy within tissue or blood. **(A)** The gating strategy for tumoral NK cells is shown for one representative patient. **(B)** The gating strategy for blood circulating NK cells is shown for one representative patient.

Supplemental Table 1 Antibodies used in this study.

## Abbreviations

EC: endometrial cancer
HD: healthy donors
LN: lymph nodes
NK: natural killer
POLE: polymerase-ε
TCGA: The Cancer Genome Atlas
Tigit: T cell Immunoreceptor with Ig and ITIM domain
Tim-3: T cell immunoglobulin and mucin domain containing 3.
