# Supplementary figures and images for: Endometrial Tumor Microenvironment Alters Human NK Cell Recruitment, and Resident NK Cell Phenotype and Function
Source: Front Immunol. 2019 Apr 26;10:877. doi: 10.3389/fimmu.2019.00877 (PMC6498896; doi:10.3389/fimmu.2019.00877)

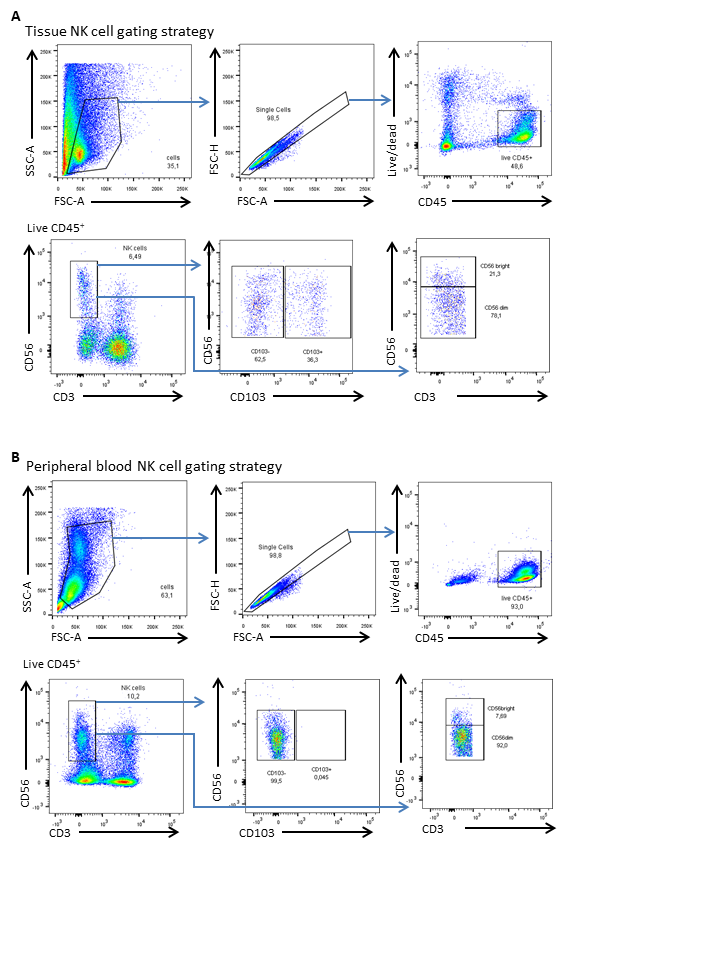

Supplement: Supplemental Figure 1 — NK cell gating strategy within tissue or blood. (A) The gating strategy for tumoral NK cells is shown for one representative patient. (B) The gating strategy for blood circulating NK cells is shown for one representative patient. [file Image_1.TIF]
